# Seaweed Slurry Improved Gel Properties and Enhanced Protein Structure of Silver Carp (*Hypophthalmichthys molitrix*) Surimi

**DOI:** 10.3390/foods11193115

**Published:** 2022-10-06

**Authors:** Liping Leng, Haotian Zou, Yingzhen Wang, Chenxu Yu, Hang Qi

**Affiliations:** 1 National Engineering Research Center of Seafood, Liaoning Provincial Aquatic Products Deep Processing Technology Research Center, School of Food Science and Technology, Dalian Polytechnic University, Dalian 116034, China; 2 Department of Agricultural and Biosystems Engineering, Iowa State University, Ames, IA 50011, USA

**Keywords:** silver carp, seaweed slurry, surimi protein, gel properties, secondary structure

## Abstract

In order to improve the gel quality of silver carp (*Hypophthalmichthys molitrix*) surimi, the texture and rheological properties and microstructure of silver carp surimi gel products were analyzed by adding *Laminaria japonica* (*LJ*), *Undaria pinnatifida* (*UP*) and *Sargassum fusiforme* (*SF*) slurries. After adding kelp slurry (KS), the gel strength reached the highest level of 1047.26 ± 85.04 (g·mm). The carbonyl content and surface hydrophobicity of surimi protein increased, while the free amino groups, total sulfhydryl groups, and fluorescence intensity decreased significantly. The results of transform infrared spectroscopy (FT-IR), circular dichroic (CD), and Raman spectra showed that the KS promoted the change of the secondary structure of protein. Scanning electron microscopy (SEM) images revealed that kelp slurry had a more compact gel microstructure. In conclusion, the kelp slurry could significantly increase the gel strength, improve the quality of surimi products, enrich the nutrition of silver carp surimi, and have great significance for expanding the application range of seaweed.

## 1. Introduction

Surimi is mainly composed of fish myofibril protein, which is made by washing away fat, blood, water-soluble protein, and other impurities [1]. Surimi products (such as fish balls and crab sticks) are popular among consumers due to their delicious taste and low price. However, the lack of marine fish resources led to a trend to utilize freshwater fish as raw material for surimi products [2]. In China, silver carp (*Hypophthalmichthys molitrix*), due to their large production, has become a potential alternative material for surimi products [3]. Due to the high yield of silver carp, the fresh fish will definitely be processed into frozen fish fillets as a reliable raw material for deep-processed fish pre-products such as surimi. However, in the processing process, different thawing and chopping processes, especially the thawing process, easily cause damage and loss of myofibrillar protein and reduce the gel strength of silver carp surimi [3]. Therefore, it is necessary to find a method to improve the gel strength of silver carp surimi and enrich its nutritional value.

Previous studies reported on four kinds of seaweed algae, green algae (*Chlorophyceae*), red algae (*Rhodophycae*), blue-green algae (*Cyanophyceae*), and brown algae (*Phaeophyceae*) [4]. Kelp (*Laminaria japonica*), wakame (*Undaria pinnatifida*) and hijiki (*Sargassum fusiforme*) are edible brown algae which are mainly grown and are distributed in Asia [5]. As brown seaweeds are rich in bioactive compounds such as polyphenols, functional polysaccharides, and other health-promoting substances, they are considered an important source of functional food [6,7]. The seaweed components are rich in ingredients such as seaweed polysaccharides; when mixing with surimi, they may assist in gelation enhancement and water holding, where seaweed components served as a stabilizer, thickener, emulsifier and gelling agent. Polysaccharides from seaweed can be used as freeze protectants for surimi products to alleviate protein denaturation [8]. Gao et al. showed that seaweed powder and sulfated polysaccharides extend the shelf life of surimi restructured products [8]. Meanwhile, several studies have demonstrated that the nutritional and textural properties of food products (meat, pasta, frankfurters, cheese) can be improved by adding the powder or extracts of seaweeds [9,10,11,12,13].

To our knowledge, several studies have been conducted on the effect of starch and protein additives on the gel properties of surimi. However, the role of seaweed slurry has not been tested. Therefore, in this study, we aimed to explore the effect of seaweed slurry on the gel properties of silver carp surimi. It is of great significance in expanding the application range of seaweed and manufacturing surimi products with improved gel strength and enriched nutrition.

## 2. Materials and Methods

### 2.1. Materials and Chemicals

Silver carp surimi (grade AA) from the same batch was purchased from Hubei Lake Modern Agricultural Technology Development Co., Ltd. (Hubei, China).

Kelp (*Laminaria japonica*, *LJ*) was provided by Shanghai Sanyuan Industrial Co., Ltd. (Shanghai, China). Wakame (*Undaria pinnatifida*, *UP*) was purchased from Dalian Liaohai Aquatic Food Trading Co., Ltd. (Dalian, China). Hijiki (*Sargassum fusiforme*, *SF*) was purchased from Wenzhou Jiahai Food Co., Ltd. (Wenzhou, China). 5 × SDS-PAGE loading buffer was purchased from Warbio Biotechnology Co. Ltd. (Nanjing, China). Bovine serum albumin (BSA), β-mercaptoethanol (β-ME), and a broad range of molecular weight markers (10–250 kDa) were purchased from Sangon Biotech Co., Ltd. (Shanghai, China). All other chemicals reagents were of analytical grade.

### 2.2. Preparation of Seaweed Blends Samples

The material of kelp (*LJ*), wakame (*UP*), and hijiki (*SF*) were soaked in water for 1 h. After getting the seaweeds out of water, the same quality of the each seaweed was mixed with water at the ratio of 1:4 (*w*/*w*). The mixture was then beat into slurry using a beater (Joyoung, Shandong, China). Among them were kelp slurry (protein, 1.74 g/100 g, polysaccharide, 12.24 g/100 g, total phenol content, 0.91 mg GAE/g). Hijiki slurry (protein, 2.46 g/100 g, polysaccharide, 10.88 g/100 g, total phenol, 2.62 mg GAE/g). Wakame slurry (protein, 3.45 g/100 g, polysaccharide, 8.12 g/100 g, total phenol, 1.98 mg GAE/g).

### 2.3. Surimi Gel Preparation

The silver carp surimi was thawed at 4 °C and chopped for 2 min using a mincer. 2.5% salt (measured by the mass of the silver carp surimi) was added, chopped, and mixed for 8 min. The surimi was divided into four parts, which were blended with 25% deionized water (control: no seaweed slurry), 25% wakame slurry (WS), 25% hijiki slurry (HS), and 25% kelp slurry (KS). Exactly 25% seaweed slurry was added, according to the results of previous pre-experiment, and chopped and mixed for another 8 min. The temperature of the whole process was controlled below 10 °C. After cutting and mixing, the mixtures were immediately placed into a tube with a diameter of 25 mm and height of about 30 mm, with both ends of the plastic wrap tied tightly. The surimi gel of silver carp was obtained by heating the mixtures at 90 °C for 30 min followed by ice water cooling at 4 °C for 30 min. The group without added seaweed slurry was set as the control group.

### 2.4. Gel Strength

The samples were determined using a texture analyzer (model TA-XT2, Stable Micro Systems, Surrey, UK). The gel samples (2.0 cm height) were placed at room temperature before testing. The speed of the probe was 1.0 mm/s. Each measurement was repeated at least six times. The gel strength was calculated as:

Gel strength (g·mm) = Breaking force (g) × Deformation (mm)(1)

### 2.5. Texture Profile Analysis

Texture profile analysis (TPA) was determined using a texture analyzer (model TA-XT2, Stable Micro Systems, Surrey, UK). The speed of the probe was 1.0 mm/s. For each sample, at least six measurements were obtained.

### 2.6. Color Measurement

The color of the gels was measured by a colorimeter (UltraScan PRO, Hunterlab Co., Ltd., Reston, VA, USA). It can be obtained (L*) brightness; (a*) indicates red-greenness, b* indicates yellow-blue, and whiteness values. The whiteness calculation formula was as follows:

Whiteness = 100 − [(100 − L*)^2^ + a*^2^ + b*^2^]^1/2^ (2)

### 2.7. Water Holding Capacity (WHC) and Cooking Loss

The water WHC of the gel samples were measured by centrifugation (Legend Micro 17R, Thermo Fisher Scientific, Waltham, MA, USA) with a slight modification [14]. First, before centrifugation, the weight of the sample was recorded as m_1_. The mass of the sample after centrifugation was then recorded as m_2_. The WHC was calculated as follows:

(3) $$\mathrm{WHC}\;(\%)=\frac{m_{2}}{m_{1}}\;\times \;100\%$$

The cooking loss was measured according to Cao & Xiong [15]. m_1_ is the weight of each gel sample. m_2_ is the weight of the sample after heating. The cooking loss was calculated as follows:

(4) $$\mathrm{Cooking}\;\mathrm{loss}\;(\%)=\frac{m_{1}{\;-\;m}_{2}}{m_{1}}\;\times \;100\%$$

### 2.8. Dynamic Rheology Measurement

The dynamic rheological properties of the samples were measured by using a rheometer with a parallel plate geometry (diameter 40 mm) (Rheometics Discovery HR-1, TA Instruments, New Castle, DE, USA) according to the method of Shi et al. [16] with some modifications. The samples were heated from 20 °C to 90 °C at a rate of 2 °C/min. The storage modulus (G′) and loss modulus (G″) changes were recorded.

### 2.9. Measurement of Structural-Related Changes of the MP

#### Amino Acid Residue Side-Chain Groups

The degree of oxidation of protein amino acid side chain groups was determined by measuring the total carbonyl, total sulfhydryl, and free amino groups of the proteins. Levine et al. reported that the 2,4-dinitrophenylhydrazine (DNPH) colorimetric method determined the carbonyl content [17].

In brief, the 200 μL samples were dissolved in 400 μL of 10 mM DNPH solution, and the mixture was placed at room temperature for 1 h under dark conditions and was shaken every 10 min. The obtained solution was added with 500 μL 20% to an ice bath for 10 min. The samples were centrifuged for 15 min (4 °C, 12,000× *g*), and the supernatant was discarded. The precipitate was washed three times with 1 mL of a mixture of anhydrous ethanol and ethyl acetate (1:1), and then the precipitate was dissolved with 250 μL of 6 M guanidine hydrochloride (dissolved in 20 mM pH 2.3 potassium phosphate buffer) in a water bath at 37 °C for 15 min. After that, the supernatant was centrifuged at 12,000× *g* for 15 min, and 200 μL of the supernatant was taken, the absorbance value at 370 nm was detected by enzyme standardization, and the carbonyl content was calculated using a molar extinction coefficient of 22,000 M^−1^ cm^−1^.

The total sulfhydryl content was tested following the procedure reported by Liu et al. [18]. Forty μL samples were dissolved in 160 μL of Tris-Gly-8 M urea solution and 1.6 μL of 4 mg/mL DTNB (dissolved in Tris-Gly buffer). Afterwards, the samples were placed in a 37 °C for 30 min in the dark and then the samples’ absorbance were read at 412 nm. The sulfhydryl content was calculated using a molar extinction coefficient of 13,600 M^−1^ cm^−1^.

Free amino content was determined according to the procedure reported by Adler-Nissen. [19]. Fifty microliter samples were dissolved in 500 μL of 0.2 M sodium phosphate buffer (pH 8.2) containing 1% sodium dodecyl sulfate (SDS), and then mixed with 250 μL of 0.01% 2,4,6-trinitrobenzene sulfonic acid (TNBS) solution. The mixed samples were reacted at 50 °C for 30 min in the dark. Afterwards, the reaction of the samples was terminated by adding 500 μL of 0.1 M Na_2_SO_3_ solution, and the samples were placed at room temperature for 15 min. After that, the absorbance of the samples at 420 nm were measured and calculated according to the standard curve generated using L-Leucine.

### 2.10. MP Secondary Structure

FTIR spectra was used to measure the wavenumber region at 400–4000 cm^−1^ according to the method of Zhou et al. [20]. Circular Dichroism (CD) was used to measure protein structure, and the test condition was 100 nm/min with a spectral range from 190 nm to 260 nm. Raman spectra was determined using 532 nm laser excitation line, with an average scanning time of 15 s and a scanning range of 900–1900 cm^−1^.

### 2.11. Fluorescence Spectrum

The fluorescence spectrum was measured by a fluorescence spectrophotometer (F-2700, Hitachi, Tokyo, Japan). The MP concentration was adjusted to 1 mg/mL. The test parameters were as follows: the excitation wavelength was 283 nm and the emission spectrum was 300–450 nm. The bandwidth used for excitation and emission was set to 5 nm.

### 2.12. Surface Hydrophobicity

Surface hydrophobicity was measured according to the method of Chelh et al. [21] with slight modification. The calculation formula of bromophenol blue (BPB) content was as follows:

(5) $$\mathrm{BPB}\;\mathrm{bound}\;(\mu g)=20\;\mu g\;\times \;\frac{A_{1}-A_{2}}{A_{1}}\;\times \;100\%$$

A_1_: blank group absorbance, A_2_: sample group absorbance.

### 2.13. Scanning Electron Microscopy (SEM)

SEM was performed as measured by Mi et al. [3] with minor modification The samples (1 × 1 × 1 mm) were immersed in 2.5% glutaraldehyde at 4 °C. Then, the samples were rinsed three times with phosphate buffer (0.2 mol/L, pH 7.0) and then dehydrated in 50%, 60%, 70%, 80%, 90% and 100% gradient ethanol (once for 15 min). Finally, scanning electron microscopy (JSM-7800F, Oxford Instruments, Abingdon, UK) was used to observe the microstructure of the freeze-dried gel sample under 10 kV.

### 2.14. SDS-Polyacrylamide Gel Electrophoresis (SDS-PAGE)

The cross-linking pattern of proteins in MP gel samples were measured following the procedure of Balange & Benjakul [22] and Leammli [23] with a slight modification. A gel consisting of 5% concentrated and 10% separating gel was added to 20 µg of the sample. After electrophoresis, staining and destaining were performed, and images were taken for further analysis.

### 2.15. Statistical Analysis

Each experiment was repeated at least in triplicate and analyzed for variance. The results were expressed as the means ± standard deviation. All data were performed by the SPSS 19.0 (SPSS Inc., 2001, Chicago, IL, USA), where *p* < 0.05 was considered the significance level.

## 3. Results and Discussion

### 3.1. Gel Strength and TPA

Gel strength and TPA results were shown in Table 1. The addition of seaweed slurry significantly increased the gel strength and TPA of surimi gels, compared with the control group (*p* < 0.05). WS, HS, and KS represent the wakame slurry, hijiki slurry, and kelp slurry, respectively. Polysaccharides, especially alginate in seaweeds, were widely applied in many food industries because of their safety and functional characteristics, especially the perfect gelling and thickening ability [24]. They may contribute good gelling properties to the surimi products. Polyphenols in seaweeds could serve as oxidizing agents and potential protein crosslinkers. They can react with side chain amino groups of peptides, leading to protein cross-linking [25]. Proteins, especially plant proteins, can not only be used as natural protease inhibitors in surimi to restrain the hydrolysis of proteins, but also form a co-gelling or act as fillers in surimi to increase gel strength [26].The KS group has the highest gel strength in all sample groups. The addition of seaweed slurry on the texture of the gel surimi system varies with the content of seaweed ingredients. The difference in gel strength depends on the content of polysaccharides, polyphenols, and proteins in the WS, HS, and KS groups. Among them, compared with WS and HS, the KS has the highest content of polysaccharides, and then the main substances in polysaccharide is alginate, laminaran, and fucoidan. The main substance in the polysaccharides is alginate, which is used in many commercial applications as thickeners, stabilizers, and emulsifiers [27]. The role of polysaccharides as a gelling agent effectively increases the connectivity of the surimi gel network, thereby increasing the gel strength. A similar result was also reported by Li, et al. who revealed that the alginate could improve the fish scale gel strength [28]. Since the seaweed slurry contains small amounts of polyphenols, it is assumed that the polyphenols play a role in the formation of the stable and dense gel structures [22]. Therefore, the KS group has the largest gel strength, which might be partly attributed to the presence of polyphenols in seaweed slurry.

The change trend of texture profiles of surimi gel products is basically consistent with the results of gel strength. Compared with the control group, the addition of seaweed slurry has significant changes in hardness, springiness, cohesiveness and chewiness (*p* < 0.05). A similar phenomenon was observed for texture profiles, which increased as KS was added. The phenomenon of aggregation and separation may be due to the interaction between alginate and protein molecules. Therefore, a composite gel was formed for the surimi gel product with seaweed slurry. This phenomenon was consistent with the results shown by Fernández-Martín et al. [29]. Chewiness and hardness significantly increased as WS, HS and KS were added (*p* < 0.05), but cohesiveness and springiness showing this increased trend is not obvious. This result is similar with the study reported by Zhang et al., who showed that Konjac glucomannan enhanced the springiness of Alaska Pollock surimi gels [30]. Moreover, the increase in springiness is due to the formation of large polymers between the additives and myofibril protein. Consequently, this result suggests that the KS could effectively improve the textural properties of surimi gel.

### 3.2. Color

As shown in Table 1, the addition of different seaweed slurries has a significant effect on the color of silver carp surimi gel. Compared with the control group, the addition of seaweed slurry led to L* and whiteness values significantly decreased (*p* < 0.05), and the b* value significantly increased (*p* < 0.05). The value of a* didn’t significantly change in all samples. It was reported that the color of gels were largely dependent on the types or color of additives [31]. Similar results were obtained by Alipour et al., who showed an increase in the b* value of surimi with the addition of polysaccharides from green alga Ulva intestinalis [32]. The color of the surimi difference of seaweed slurry groups mainly depends on the color of additives.

### 3.3. WHC and Cooking Loss

**Figure 1:**
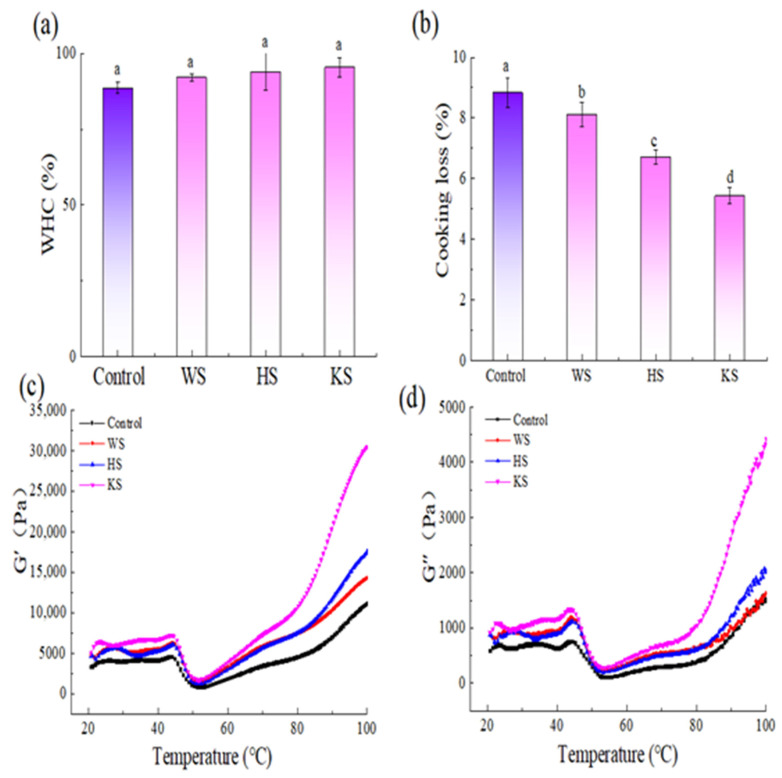
Gel properties (**a**,**b**) and rheological properties (**c**,**d**) of silver carp surimi with different treatments. Control: Surimi (75%) + water (25%); WS: Surimi (75%) + wakame slurry (25%); HS: Surimi (75%) + hijiki slurry (25%); KS: Surimi (75%) + kelp slurry (25%). Values are expressed as the means ± SD and results are representative of at least three independent experiments. a–d indicates that the different letters are significantly different (*p* < 0.05).

WHC is an important quality parameter of surimi food. The changes in WHC of samples with the addition of seaweed slurry are shown in Figure 1a. The WHC of the WS, HS, and KS groups were not significantly increased compared with control group. However, the WHC of the KS group is larger than the WS and HS group. The reason for the highest WHC of the KS group may be that the KS contains more alginate than WS and HS. The researchers infer that the hydrophilicity of sodium alginate and the ability to promote the formation of network gels, and lead to improve WHC [33,34]. It is speculated that with the increase of alginate content, more sodium alginate/water and sodium alginate/meat protein interactions will occur in the gel. Therefore, the KS group has the best WHC capacity. The reason why the WHC of the HS group is higher than WS group may be attributed to the difference in polyphenol content. A similar report was obtained by Balange & Benjakul [22], who revealed that the addition of phenolic compounds can enhance the WHC of mackerel surimi.

Figure 1b showed the changes in cooking loss of samples containing seaweed slurry. The cooking loss values of the WS, HS, and KS group were significantly increased (*p* < 0.05) compared with control surimi gel (*p* < 0.05). The KS group has the lowest cooking loss with the addition of KS among all groups. This result was consistent with WHC. The main reason might be that the KS contained a lot of alginate. After the alginate is combined with water molecules, which will fill the gaps of the surimi gel protein network, they form a more compact gel microstructure [28]. Therefore, KS can significantly reduce the cooking loss of surimi gel.

### 3.4. Dynamic Rheological Measurements

The storage modulus (G′) and loss modulus (G″) of all samples at 20 to 100 °C are depicted in Figure 1c,d. Compared with the control group, the G′ and G″ of the samples were both increased with the addition of seaweed slurry. Among them, the KS group has the highest G′, and the HS group G′ is higher than the WS group. The G′ value reflects the elastic properties of the sample. For all samples, the patterns of G′ were always higher than G″. This indicates that the sample had higher elasticity. Generally, G′ would reach the maximum of 45 °C when heated from 20 °C to 45 °C. When the sample was heated from 45 °C to 50 °C, G′ dropped and reached a minimum value at about 50 °C. It corresponded to the formation of disulfide bonds of the myosin head and the aggregation of uncoiled myosin tails [16,35]. After samples were heated from 50 °C to 100 °C, the G′ and G″ rapidly increased, and the KS group reached the maximum. This could be attributed to the addition of KS, which contained alginate that further inhibited the formation of disulfide bonds of the myosin head, resulting in myosin gelation. This result was in agreement with the study reported by Shi et al., who showed that ultrasound-assisted potassium alginate could increase the gel properties [16]. Meanwhile, the KS group showed a higher final G′ value than the WS and HS groups with the increase of temperature, which was beneficial in improving the gel strength.

### 3.5. Structural Changes

#### Amino Acid Residue Side-Chain Groups

Carbonyl groups. The degree of protein carbonylation is a recognized indicator of protein oxidation. As shown in Table 2, compared with the control sample, the carbonyl content in seaweed slurry groups significantly increased (*p* < 0.05). Utrera & Estevez reported that the production of carbonyl groups was effected by phenol, which depends on the concentration of phenol and other conditions [36]. They indicated that the seaweed slurry can slightly increase the production of carbonyl. Our results were consistent with previous research results studied by Jiang et al. [37]. Furthermore, the increase in the carbonyl content of the added seaweed slurry was affected by multiple components which might be the combination of polyphenols with proteins. Our results showed that KS could increase the carbonyl content.

Sulfhydryl groups. The sulfhydryl group is considered the most active group. The functional groups in proteins have significant influence on the functional properties of food proteins [38]. As observed in Table 2, compared with control sample, the sulfhydryl content in seaweed slurry groups significantly decreased (*p* < 0.05). Cao, et al. showed that phenols were easily oxidized to form quinones, which can form sulfhydryl-quinone addition products with sulfhydryl groups [39]. Therefore, the decrease in sulfhydryl content was probably due to the covalent cross-linking of polyphenols with the sulfhydryl groups. Feng et al. found that EGCG promotes the reduction of myofibrillar protein sulfhydryl content, which was also due to the formation of sulfhydryl-quinone addition products [40]. However, Gao et al. found that the total sulfhydryl content of surimi with soluble soybean polysaccharide decreased slightly [8]. Therefore, the degree of sulfhydryl content decrease is related to the type and content of polyphenols and polysaccharides. In this study, our result indicated that KS could significantly reduce the sulfhydryl content.

Free amino groups. As observed in Table 2, compared with the control group, the free amino contents in seaweed slurry groups significantly decreased (*p* < 0.05). The reason might be that the amino group can also be combined with the formed quinone compound, resulting in the reduction of the free amino group in MP. Chen & Sun showed that most of the reduction of free amino groups in protein is due to the oxidation of protein [41]. However, the reduction of amino groups between HS and KS groups were not significant (*p* < 0.05). The result was consistent with the study reported by Li et al. [42]. This result showed that the KS could significantly reduce the free amino content.

### 3.6. Secondary Structure

**Figure 2:**
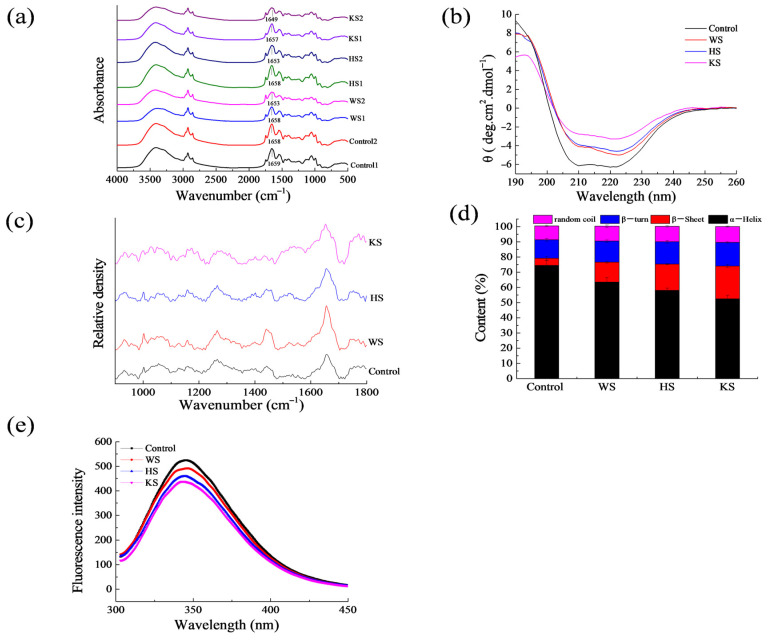
Changes in structure of myofibrillar protein (MP) with different treatments. (**a**): FTIR spectra; (**b**): Circular dichroism; (**c**): Raman spectra (**d**): Raman spectra secondary structure content; (**e**): Fluorescence intensity; Control: Surimi (75%) + water (25%); WS: Surimi (75%) + wakame slurry (25%); HS: Surimi (75%) + hijiki slurry (25%); KS: Surimi (75%) + kelp slurry (25%); Values are expressed as the means ± SD and results are representative of at least three independent experiments.

FTIR spectra. Figure 2a displayed the FTIR spectra of surimi protein modified by different seaweed slurry. The samples have several characteristic absorption peaks in the infrared region which can reflect the changing trend of protein secondary structure. Among them, the amide I absorption (1700–1600 cm^−1^) represents the stretching vibration of the C=O bond and was used for the determination of the protein’s secondary structure [43]. Control1, WS1, HS1, and KS1 represents surimi protein treated with different seaweed slurries. Control 2, WS2, HS2, KS2 represent the gelatinized surimi protein treated with seaweed slurries. During the gelation process, the absorption peak reflecting the characteristic of α-helix in surimi shifted to a low wave number. The results showed that the α-helix of surimi protein added with seaweed slurry was transformed into a β-sheet structure during the gelation process, which was consistent with the subsequent CD chromatography and Raman spectroscopy results. Zhou et al. demonstrated a similar result of EW protein structural changes obtained by tea polyphenols [20].

CD. AS shown in (Figure 2b, Table 3). CD spectroscopy showed a decrease in the α-helix content of the samples with the addition of seaweed slurry, while the contents of β-sheet, β-turn and random coils did not show significant changes (Figure 2b, Table 3). Compared with the control sample, there were no significant differences in protein secondary structure between the WS and HS groups (*p* > 0.05). The addition of WS and HS had no obvious effect on the surimi protein’s secondary structure. However, compared with the control group, the content of the α-helix structure obviously decreased, while the content of β-sheet, β-turn and random coils obviously increased in the KS group (*p* < 0.05). The study showed that KS significantly changed the protein conformation of surimi, which was conductive to the interaction between protein and alginate. This is because the KS contains a lot of alginate. The combination of alginate and surimi will produce salt bonds and form polyelectrolyte composite. Meanwhile, the alginate contains a large number of polar groups, which will interact with proteins to form a large number of hydrogen bonding effects. Under the combined action of hydrogen bonds, the secondary structure of the protein gradually changes from α-helix to β-sheet, and finally reduces the content of α-helix, thereby significantly increasing the content of β-sheet [44]. In addition, Jia et al. also reported that forming β-LG− EGCG complexes changed the β-LG secondary structure inducing α-helix to β-structures transition, which was consistent with the FTIR results [45]. To summarize, the KS could significantly change the protein structure.

Raman spectra. Figure 2c showed the Raman experiment of different treatments of seaweed slurry in the wavenumber range of 900–1800 cm^−1^ in MPs. The Amide I band mainly involves C=O stretching, located in the range of 1650–1660, 1660–1665, 1665–1680, and close to 1680 cm^−1^, which are representative of α-helix, random coil, β-sheets and β-turn structures, respectively [46]. The Amide I band can be used to quantitatively analyze the content of the MP secondary structure [47]. The Raman curves of different seaweed slurry and the percentage content of the secondary structure are shown in Figure 2c,d. Compared with the control group, the groups added with seaweed slurry had significant changes in the contents of α-helix and β-sheet (*p* < 0.05), but not β-turn and random coil (*p* > 0.05). Among them, the content of α-helix had a significant decrease in the KS group, while the β-sheets were markedly decreased. This result was consistent with the study reported by Yang et al., who demonstrated that the decrease of α-helix and the increase of β-sheet within the 7S protein existed with the addition of EGCG [48]. Previous researchers have also shown that β-sheet formation is favorable for gelling, and the content of the β-sheet structure is positively correlated with the hardness of surimi gel [14,49].

### 3.7. Fluorescence Spectrum

Fluorescence spectrum. As observed in Figure 2e, the fluorescence intensity of the control group was the highest among all groups, and the maximum peak was 345.25 nm. Compared with the control sample, the highest values of fluorescence intensity in the WS, HS, and KS group were 346.25 nm, 344.75 nm and 343.25 nm, respectively. With the addition of seaweed slurry, a decrease trend of MP fluorescence intensity was observed. The decrease of fluorescence intensity is usually due to the quenching of the endogenous tryptophan by another substance (e.g., polyphenols). It was reported that polyphenols bind to aromatic amino acid residues on the protein surface, resulting in the reduced fluorescence intensity effect of another substance (such as polyphenols) on the aromatic amino acids [50]. This result was similar to the study reported by Parolia et al., who reported that lentil protein isolate conjugated with plant polyphenols (quercetin, rutin, ellagic acid) resulted in an appreciable decrease in fluorescence intensity [51]. In addition, Hu et al. concluded that the fluorescence intensity of *Pleurotus eryngii* polysaccharide conjugate soy protein isolate was reduced [52]. The lowest fluorescence intensity was observed in the samples containing KS, indicating that the polyphenols and polysaccharide in kelp combined with the aromatic amino acid residues on the protein surface, resulting in the decrease of fluorescence intensity.

### 3.8. Surface Hydrophobicity

As shown in Table 2, the hydrophobicity on the surface of surimi protein with seaweed slurry significantly increased compared to the control group (*p* < 0.05). The surface hydrophobicity of the KS group was improved by 47.4% compared with the control group. Jiang et al. found the same trend in surface hydrophobicity that was based on the polyphenols interacting with the protein, further changed the conformation, and increased the surface hydrophobicity [53]. Usually the increase in surface hydrophobicity is due to the unfolding of the protein structure and exposing the hydrophobic residues buried in the interior of the protein [54]. In addition, Perez et al. showed that as the concentration of sodium alginate increases, the surface hydrophobicity of milk whey protein was increased [55]. In the present result, it is speculated that the interaction between the protein and the polysaccharide causes the protein to unfold, resulting in a higher exposure of the protein hydrophobic fragments protein. That is because of the different content of seaweed polyphenols and polysaccharides in seaweed. Therefore, the KS can significantly increase the surface hydrophobicity of surimi protein.

### 3.9. Microstructure of Surimi Gel

**Figure 3:**
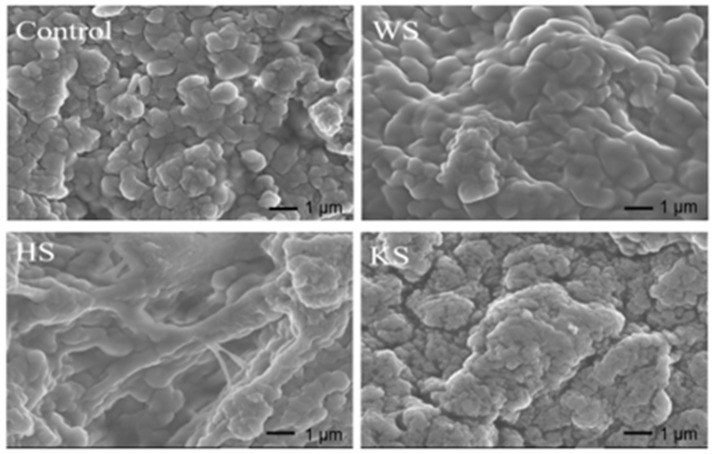
SEM images of surimi gel (10.0 k) with different treatments. Control: Surimi (75%) + water (25%); WS: Surimi (75%) + wakame slurry (25%); HS: Surimi (75%) + hijiki slurry (25%); KS: Surimi (75%) + kelp slurry (25%).

The three-dimensional network structure of a gel is an important determinant of its functional properties. As observed in Figure 3, the control group had coarser and larger pores in the gel structure, while the surimi gel with seaweed slurry revealed a denser smooth structure and uniform structure, although there were some holes (Figure 3). Among them, the microstructure of gel with KS group was more compact and denser. This is mainly due to the different content of polysaccharides and polyphenols in the seaweed slurry, etc. Polysaccharides could make the surimi gel form a dense surface structure. Moreover, the increase in polysaccharide content leads to the formation of more aggregates. Therefore, the KS group formed agglomerates (Figure 3). This result was in agreement with the study of Li et al. who reported that the excessive addition of polysaccharides in FS gels can cause changes in the microstructure [28]. The changes in the microstructure of the gel were caused by the formation of aggregates during the formation of the gel [56]. Zhou et al. reported that tea polyphenols could enhance the compactness of the protein network [20]. The polyphenol might be another factor to make the gel structure more dense compared with other groups due to the protein crosslinkers function. Therefore, it strongly demonstrated that KS can make the surimi form a denser, continuous and uniform gel.

## 4. Conclusions

In conclusion, the KS group was more advantageous for improving surimi gel properties of surimi than the WS group and the HS group. The KS group had a significant increase in the gel strength and texture profile of silver carp surimi gel compared to the WS group and the HS group. Meanwhile, the KS group increased storage modulus (G′) and loss modulus (G″) showed a denser and uniform surimi gel surface structure. Furthermore, FT-IR, CD, and Raman spectra showed that the secondary structure was obviously changed. Hence, the KS group had effective surimi gel properties in surimi products. In the future, we will further analyze which components in seaweed have important effects on protein gel properties. We hope the present study could expand the utilization of brown seaweeds in the food processing field.

## Figures and Tables

**Table 1:** Color and texture of silver carp surimi gel with different treatments.

	Control	WS	HS	KS
Gel strength (g·mm)	403.41 ± 51.82 ^d^	475.09 ± 42.89 ^c^	629.52 ± 64.84 ^b^	1047.25 ± 85.04 ^a^
Breaking force (g)	84.19 ± 8.89 ^c^	100.81 ± 6.04 ^bc^	119.59 ± 11.93 ^b^	162.36 ± 21.74 ^a^
Breaking distance (mm)	4.78 ± 0.16 ^b^	4.71 ± 0.15 ^b^	5.26 ± 0.03 ^b^	6.52 ± 0.97 ^a^
Hardness (g)	832.28 ± 50.06 ^c^	1121.73 ± 50.06 ^b^	1183.55 ± 51.09 ^b^	1712.34 ± 70.69 ^a^
Springiness	0.67 ± 0.07 ^b^	0.78 ± 0.05 ^a^	0.81 ± 0.03 ^a^	0.84 ± 0.01 ^a^
Cohesiveness	0.41 ± 0.05 ^c^	0.54 ± 0.06 ^b^	0.60 ± 0.01 ^ab^	0.69 ± 0.07 ^a^
Chewiness	316.51 ± 20.25 ^d^	508.06 ± 44.98 ^c^	588.31 ± 43.89 ^b^	863.60 ± 41.05 ^a^
Lightness (L*)	77.49 ± 1.29 ^a^	65.26 ± 2.74 ^c^	50.64 ± 2.37 ^d^	70.77 ± 1.51 ^b^
Redness (a*)	−2.76 ± 0.02 ^b^	−5.31 ± 0.27 ^c^	3.09 ± 0.76 ^a^	−2.88 ± 0.10 ^b^
Yellowness (b*)	4.25 ± 0.33 ^c^	10.95 ± 0.50 ^a^	9.63 ± 0.66 ^b^	9.41 ± 0.89 ^b^
Whiteness	76.93 ± 1.31 ^a^	63.18 ± 2.68 ^c^	49.61 ± 2.38 ^d^	69.15 ± 1.40 ^b^

Control: Surimi (75%) + water (25%); WS: Surimi (75%) + wakame slurry (25%); HS: Surimi (75%) + hijiki slurry (25%); KS: Surimi (75%) + kelp slurry (25%); Values are expressed as the means ± SD and results are representative of at least three independent experiments. a–d indicates that the different letters are significantly different (*p* < 0.05).

**Table 2:** Physicochemical changes of surimi protein with different treatments.

Treatment	Carbonyl (nmol/mg Protein)	Total Sulfhydryl (nmol/mg Protein)	Free Amino (nmol NH_2_/mg Protein)	BPB Bound
Control	0.93 ± 0.02 ^d^	32.72 ± 1.80 ^a^	0.118 ± 0.020 ^a^	7.48 ± 0.59 ^c^
WS	1.14 ± 0.02 ^c^	26.34 ± 2.81 ^b^	0.079 ± 0.007 ^b^	8.47 ± 0.56 ^b^
HS	1.56 ± 0.10 ^b^	23.07 ± 2.72 ^bc^	0.028 ± 0.003 ^c^	8.83 ± 0.25 ^b^
KS	1.79 ± 0.10 ^a^	19.07 ± 0.89 ^c^	0.024 ± 0.001 ^c^	11.1 ±0.51 ^a^

Control: Surimi (75%) + water (25%); WS: Surimi (75%) + wakame slurry (25%); HS: Surimi (75%) + hijiki slurry (25%); KS: Surimi (75%) + kelp slurry (25%); MP: myofibrillar protein. BPB: bromophenol blue (as a measure of protein surface hydrophobicity). Values are expressed as the means ± SD and results are representative of at least three independent experiments. a–d indicates that the different letters are significantly different (*p* < 0.05).

**Table 3:** Relative contents of secondary structural elements in surimi protein.

Treatment	α-Helix (%)	β-Sheet (%)	β-Turn (%)	Random (%)
Control	54.00 ± 1.4 ^a^	1.07 ± 0.21 ^b^	17.10 ± 0.82 ^b^	27.83 ± 1.78 ^b^
WS	53.00 ± 1.20 ^a^	0.00 ± 0.00 ^b^	17.83 ± 0.32 ^b^	29.17 ± 1.40 ^b^
HS	51.43 ± 0.26 ^a^	0.00 ± 0.00 ^b^	20.20 ± 0.44 ^a^	28.37 ± 0.15 ^b^
KS	36.37 ± 0.35 ^b^	12.93 ± 1.42 ^a^	15.97 ± 0.49 ^c^	34.73 ± 0.75 ^a^

Control: Surimi (75%) + water (25%); WS: Surimi (75%) + wakame slurry (25%); HS: Surimi (75%) + hijiki slurry (25%); KS: Surimi (75%) + kelp slurry (25%); Values are expressed as the means ± SD and results are representative of at least three independent experiments. a–c indicates that the different letters are significantly different (*p* < 0.05).

## Data Availability

The data presented in this study are available on request from the corresponding author.
